# c-Jun N-terminal kinase 2 suppresses pancreatic cancer growth and invasion and is opposed by c-Jun N-terminal kinase 1

**DOI:** 10.1038/s41417-020-00290-5

**Published:** 2021-02-01

**Authors:** Xiaodong Tian, Benno Traub, Jingwei Shi, Nadine Huber, Stefan Schreiner, Guowei Chen, Shaoxia Zhou, Doris Henne-Bruns, Uwe Knippschild, Marko Kornmann

**Affiliations:** 1 Clinic for General and Visceral Surgery, Ulm, Germany; 2 Clinical Chemistry, University of Ulm, Ulm, Germany

**Keywords:** Pancreatic cancer, Cancer genetics

## Abstract

The c-Jun N-terminal protein kinases (JNKs) JNK1 and JNK2 can act as either tumor suppressors or pro-oncogenic kinases in human cancers. The isoform-specific roles for JNK1 and JNK2 in human pancreatic cancer are still unclear, the question which should be addressed in this project. Human pancreatic cancer cell lines MIA PaCa-2 and PANC-1 clones were established either expressing either JNK1 or -2 shRNA in a stable manner. Basal anchorage-dependent and –independent cell growth, single-cell movement, and invasion using the Boyden chamber assay were analyzed. Xenograft growth was assessed using an orthotopic mouse model. All seven tested pancreatic cancer cell lines expressed JNKs as did human pancreatic cancer samples determined by immunohistochemistry. Pharmacological, unspecific JNK inhibition (SP600125) reduced cell growth of all cell lines but PANC-1. Especially inhibition of JNK2 resulted in overall increased oncogenic potential with increased proliferation and invasion, associated with alterations in cytoskeleton structure. Specific inhibition of JNK1 revealed opposing functions. Overall, JNK1 and JNK2 can exert different functions in human pancreatic cancer and act as counter players for tumor invasion. Specifically modulating the activity of JNKs may be of potential therapeutic interest in the future.

## Introduction

Human pancreatic cancer is an aggressive and devastating malignancy with a poor prognosis. Due to invasive growth, early distant metastasis, frequent recurrence after surgical resection, and resistance to chemo- and radiotherapy, the average overall 5-year survival rate is still only 9% despite improvements in surgical and chemotherapeutic approaches [1, 2]. Intensive research during the past decades revealed multiple dysfunctions of several oncogenes, tumor suppressor genes, and growth factor pathways involved in its pathobiology [3, 4].

The c-Jun N-terminal protein kinases (JNKs), which were also referred to as stress-activated kinases (SAPKs) for their activation in response to cell stress such as UV irradiation, are members of the mitogen-activated protein kinase (MAPK) family. In mammalian genomes, three JNK genes have been identified. JNK1 and JNK2 are ubiquitously expressed in tissues, while JNK3 expression is mainly restricted to the brain, heart, and testes [5]. At least ten transcripts are formed by alternative splicing to create 46 and 55 kDa proteins [6].

JNKs have been recognized as critical regulators for several physiological functions like cell proliferation, apoptosis, DNA repair, and metabolism. The variety of different physiologic functions is addressed by multiple downstream substrates, among those c-Jun, ATF-2, and p53 (ref. [7]). Expectedly, within this complexity, there is still room for opposing functions like inducing apoptosis as well as enhancement of cell survival and proliferation [8, 9].

Following this, the role of JNKs in human cancers is still unclear and discussed controversially [10]. The cellular response to JNK-stimulation seems to be dependent on cell type as well as the strength and duration of JNK-stimulation. Hereby, JNK-1 induced apoptosis can lead to tumor suppression on the one side, while c-Jun regulated malignant transformation, induced by Ras-oncogene, acts as a tumor promoter [9, 11–13]. The knowledge that pancreatic cancer harbors frequent Ras-mutations suggests JNK and c-Jun as a potential therapeutic target. This is supported by previous observations that inhibition of JNK decreased the growth of murine pancreatic cancer [14] and abolished the self-renewal and tumor-initiating capacity of pancreatic cancer stem-like cells [15]. Unexpectedly and in contrast to these findings, our group has previously shown that the over-expression of fibroblast-growth-factor-receptor-1-IIIb (FGFR1-IIIb), associated with marked up-regulation of JNK activity, inhibits the malignant phenotype of pancreatic cancer cells [16].

Presently the exact functions of JNK1 and JNK2 in the pathogenesis of human pancreatic cancer are still unclear. The aim of the present study was to separately investigate possible functions of JNK1 and JNK2 in human pancreatic cancer cells in vitro and in vivo.

## Materials and methods

### Cell culture

AsPC-1, BxPC-3, Capan-1, MIA PaCa-2, and PANC-1 human pancreatic cancer cells were purchased from American Type Culture Collection (ATCC, Manassas, VA), COLO357 and T3M4 human pancreatic cancer cells were a gift from M. Korc (Indianapolis, IN) without STR genotyping. PANC-1, MIA PaCa-2, COLO357 cells were grown in DME medium and AsPC-1, BXPC-3, CAPAN-1, T3M4 cells in RPMI 1640 medium supplemented with 10% fetal bovine serum (FCS), penicillin G (100 units/ml), and streptomycin (100 µg/ml) termed complete medium. DME and RPMI medium supplemented with 0.1% bovine serum albumin, 5 mg/L transferrin, 5 µg/l selenium, penicillin G (100 units/ml), and streptomycin (100 µg/ml) was used as a serum-free medium. Cells were maintained in monolayer culture at 37 °C in humidified air with 5% CO_2_. Regular mycoplasma testing was performed.

### Human tumor tissue

For patients who undergo curative resection for pancreatic adenocarcinoma at the Department of General and Visceral Surgery at the University of Ulm, Germany, tumor tissue is collected during surgery, provided written informed consent before tissue collection. Ten of these patients, resected between 2003 and 2012, were randomly selected and screened for JNK1 and −2 expression. None of the patients received neoadjuvant treatment. The study involving human tissues was approved by the Ethics committee of the University of Ulm (# 211/2002).

### Establishment of cell clones expressing JNK1 and JNK2 shRNA

Validated SureSilencing human JNK1, JNK2 shRNA, and control plasmids were obtained from SuperArray Bioscience Corp. (Frederick, MD). MIA PaCa-2 and PANC-1 cells were transfected in a stable manner using lipofectamine (Invitrogen, Carlsbad, CA) following the manufacturer’s protocol. MIA PaCa-2 transfected cells were selected with G418 (1.2 mg/ml) for 14 days before isolation of individual clones. PANC-1 cells were selected similarly with Puromycin (2 µg/ml) [17].

### Immunoblot analysis

Total cell lysates were prepared and followed by immunoblot analysis [18]. Rabbit anti-human JNK1 and JNK2 polyclonal antibodies (sc-571 and sc-572 for JNK1 and JNK2, respectively, from Santa Cruz (Santa Cruz, CA)) were used (1:2000 and 1:1000, respectively) to detect JNK1 and JNK2 protein. Bound antibodies were visualized using enhanced chemiluminescence. To confirm equal loading, membranes were stripped for 30 min at 50 °C in buffer containing 2% sodium dodecyl sulfate, 62.5 mM Tris (pH 6.7), and 100 mM 2-mercaptoethanol and reprobed with an anti-ß-actin antibody to demonstrate equal loading.

### JNK activity assay

To determine JNK activity, a JNK assay kit (#9810) was used according to the protocol of the manufacturer (Cell Signaling Technology, Inc. Danvers, MA). In brief, total cell lysates (250 μg in 250 μl of lysis buffer) were incubated for 20 h at 4 °C with resuspended c-Jun fusion protein beads (20 μl). After washing twice with 500 μl of ice-cold lysis buffer and kinase buffer, respectively, the pellet was resuspended in 50 μl of kinase buffer supplemented with 100 μmol/L ATP. After incubating for 30 min at 30 °C, immunocomplexes were captured by centrifugation and subjected to immunoblot analysis using phospho-c-Jun specific antibodies. Results were quantified with ImageJ V1.32 (NIH, Bethesda, MD) and displayed in relationship to JNK WT cells as mean ± SD of three independent experiments.

### Anchorage-dependent growth assay

The effects of the exogenous JNK inhibitor SP600125 (El-305 from Biomol) on the proliferation of cultured human pancreatic cancer cell lines were determined by the 3-(4,5-dimethylthiazol-2-yl)-2,5-diphenyltetrazolium bromide (MTT) assay [18]. Cells (5000 per well) were propagated for 24 h in complete medium and then incubated for another 48 h in the absence or presence of an increasing concentration of SP600125 before initiation of the MTT assay.

To assess the basal growth of different clones expressing JNK1 or JNK2 shRNA, 10,000 cells (MIA PaCa-2) or 5000 cells (PANC-1) per well were incubated for 48 h in complete medium before analysis.

### Anchorage-independent growth assay

Basal anchorage-independent cell growth was assessed by a double-layer soft agar assay as described for MIA PaCa-2 (ref. [17]). Briefly, cells were suspended in complete medium containing 0.3% agar and seeded in triplicate in 6-well plates onto a base layer of complete medium containing 0.5% agar. One microlitre of complete medium containing 0.3% agar was added every 5 days. After 14 days, 300 μg MTT/well was added to stain vital colonies for 24 h before counting by microscopy. PANC-1 showed a tendency to migrate and spread on the base layer and was thus cultured in growth-factor reduced Matrigel (Corning, Inc., Corning, NY) with 1000 cells in domes of 50 µl. PANC-1 was cultured in Matrigel for 10 days before evaluation. Two pictures were taken at 10× magnification per dome and colony size was measured for at least five colonies. MTT (250 µg/dome) was added afterward to evaluate total colony size and number (represented by overall viable cells). After incubation for 4 h, acidic isopropanol (200 µl per dome) was added photometric absorption at 570 nm was measured.

### Single-cell movement assay

Cells (50,000 per well) were seeded onto fibronectin-coated (5 µg/mL in PBS) six-well plates and grown for 20 h. Cell movement during the following 24 h was monitored by an Olympus IX81 motorized inverted microscope taking pictures every 10 min. The total distance of individual cells covered within 24 h was determined using ImageJ.

### Cell migration assay

The ability of cells to migrate through filters was measured using a BioCoat Matrigel invasion chamber (BD Biosciences, San Jose, CA USA). Cell culture inserts with an 8 µm pore size PET membrane were used according to the protocol of the manufacturer. The bottom chamber included medium (0.75 mL) containing 10% FCS, while cells (5 × 10^4^ (MIA PaCa-2) or 2.5 × 10^4^ (PANC-1)) suspended in 0.5 mL of medium containing 1% FCS) were seeded into the upper chamber and incubated 36 h at 37 °C in a humidified atmosphere containing 5% CO_2_. The remaining cells on the upper surface were mechanically removed. Membranes were then washed, fixed, and stained by Diff-Quik (Medion Diagnostics International Inc.). The number of cells that migrated to the lower surface of the filters was determined by counting stained cells under a light microscope as described [16].

### Immunohistochemistry

Expression of JNK1 and -2 was determined in specimens of patients with pancreatic ductal adenocarcinoma, who underwent tumor resection at our department. Specific recombinant rabbit monoclonal antibodies were used for JNK1 (ab110724, Abcam) and JNK2 (ab76125, Abcam). Immunohistochemistry was performed using formalin-fixed and paraffin-embedded 5 µm sections in dilution 1:250 in combination with a secondary biotinylated goat anti-rabbit antibody and a Vectastain Elite ABC kit (Vector Lab, Burlingame, CA) according to the protocol of the manufacturer. For immunohistochemical analysis of orthotopic tumors, the following antibodies were used according to the protocols of the manufacturers: Ki67 (clone SP6, Thermo Fischer Scientific, Fremont, CA), p-c-Jun (54B3, Cell Signaling Technologies), MMP-2 (SAB2108458, Sigma-Aldrich, St. Louis, MO), MMP-9 (ITA1401, G-Biosciences, St. Louis, MO), Vimentin (D21H3, Cell Signaling Technologies). All samples were collected immediately during the operation with prior obtained informed consent from all patients.

### Orthotopic xenograft model

To assess the effect of JNK1 and JNK2 expression on xenograft formation and growth, an orthotopic pancreatic cancer model was used [19]. Eight weeks old, athymic female (nude) mice were anesthetized with a ketamine-xylazine solution followed by a small midline abdominal incision. Indicated cells (2 × 10^6^) were resuspended in 50 µl PBS including 1% growth factor reduced Matrigel (BD Biosciences, Heidelberg, Germany) and injected into the subcapsular region of the pancreas using a 30-gauge needle and a calibrated push button-controlled dispensing device. The viability of the injected cells was >95% (trypan blue test) and cells were mycoplasma free. A cotton swab was held for 1 min gently on the site of injection to prevent leakage. The abdominal wound was closed in one layer with 4.0 polysorbate sutures.

Animals were monitored daily, weight was documented every other day, and mice were sacrificed 18 days after the operation. The abdomen was re-opened and macroscopically checked for tumor growth and metastatic sites. Tumor size was measured in three dimensions. Tumor volume was determined unblinded by the equation: Volume = l*w*d*0.5, where *l* is the length, *w* is the width, and *d* is the diameter. Abdominal organs were removed, formalin-fixed, and paraffin-embedded. Five-micrometer sections were prepared and used for H&E-staining and immunohistochemistry.

Mice were housed and handled according to the official regulations for the care and use of laboratory animals (UKCCCR Guidelines for the Welfare of Animals in Experimental Neoplasia). Ethical approval of the animal experiments was granted by the Regierungspräsidium Tübingen (permission number 1034).

### Statistics

Results are presented expressed as mean (±SD) and range as indicated. Group differences were assessed by means of the Student’s *t* test. If the assumption of normality distribution was not met, the nonparametric Mann–Whitney Rank Sum test was used instead. Fisher’s exact test was within the orthotopic xenograft model. A *p* value < 0.05 was taken as a level of significance (two-sided). Animal groups were analyzed non-randomized as no therapeutic intervention was performed.

## Results

### Expression of JNKs in human pancreatic cancer cells and the effect of SP600125 on in vitro pancreatic cancer cell proliferation

**Fig. 1:**
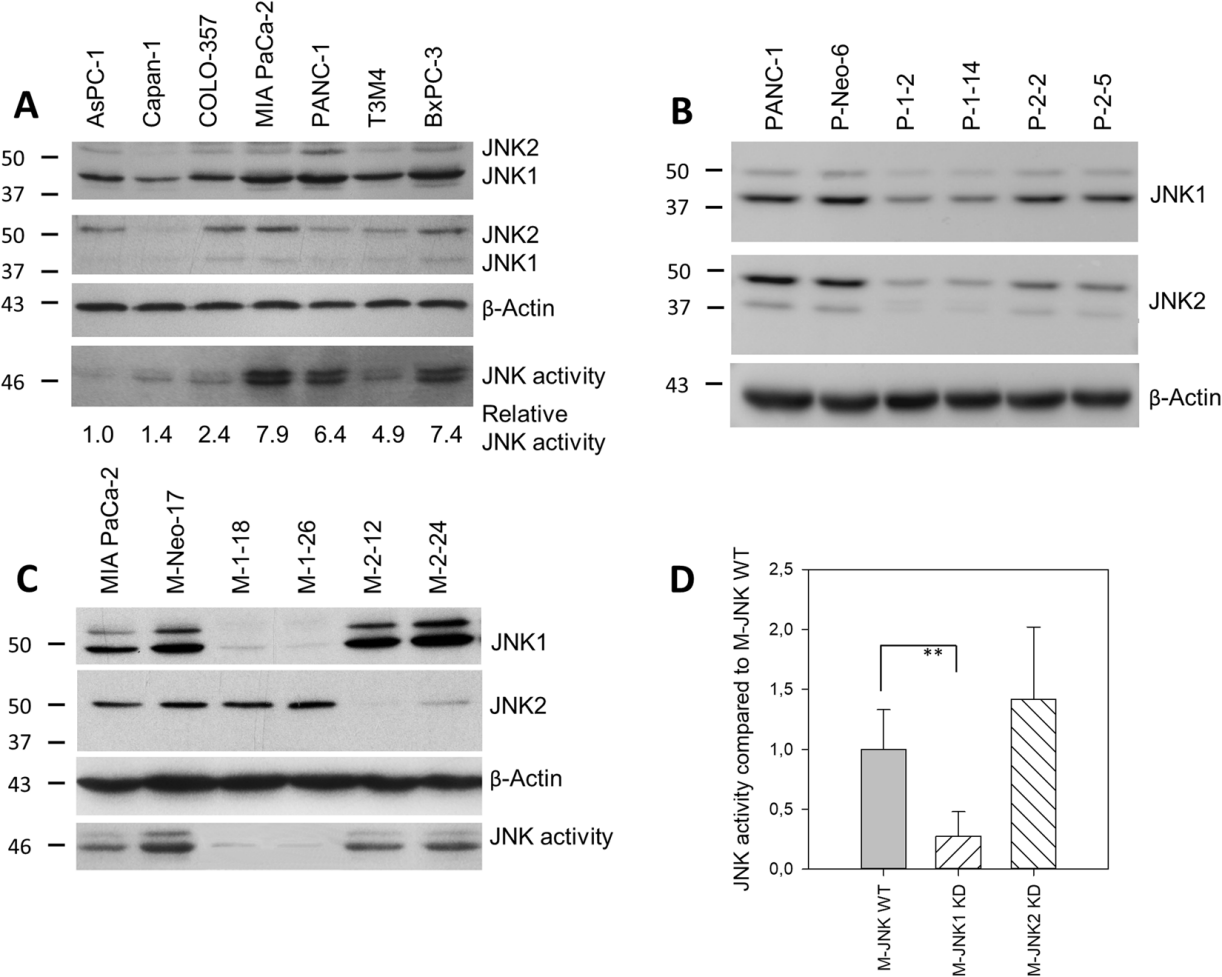
Expression and activity of JNK1 and JNK2 in pancreatic cancer cell lines. **A** JNK1 and JNK2 protein expression in seven cultured human pancreatic cancer cell lines. Western Blot was performed using JNK (sc-571) antibody primarily detecting JNK1, but cross-reactive with JNK2 (anti-JNK1) and JNK (sc-572) antibody primarily detecting JNK2, but cross-reactive with JNK1 (anti-JNK-2). β-Actin served as a loading control (third panel). Total JNK activity was determined by assessing the amount of phosphorylated c-Jun (p–c-Jun, lower panel) in a nonradioactive in vitro kinase assay (*n* = 1). Blots were scanned and relative JNK activity determined in relation to AsPC-1 cells. **B**, **C** Selective inhibition of JNK1 and JNK2 in PANC-1 (**B**) and MIA PaCa-2 (**C**). Cells were transfected in a stable manner with control shRNA (M-Neo-17/P-Neo-6), JNK1 shRNA (M-1–18, M-1–26/P-1–2, P-1–14), and JNK2 shRNA (M-2–12, M-2–24/P-2-2, P-2–5). The screening was performed using JNK antibodies (upper panels). β-Actin served as a loading control (third panel). **C**, **D** Total JNK activity was determined in MIA PaCa-2 by assessing the amount of phosphorylated c-Jun. A representative blot is shown in (**C**) and quantification of three independent experiments is shown in (**D**). JNK WT cells (WT and Neo clones), JNK1 KD clones, and JNK2 KD clones were analyzed together. **p < 0.001 compared to JNK WT cells.

**Fig. 2:**
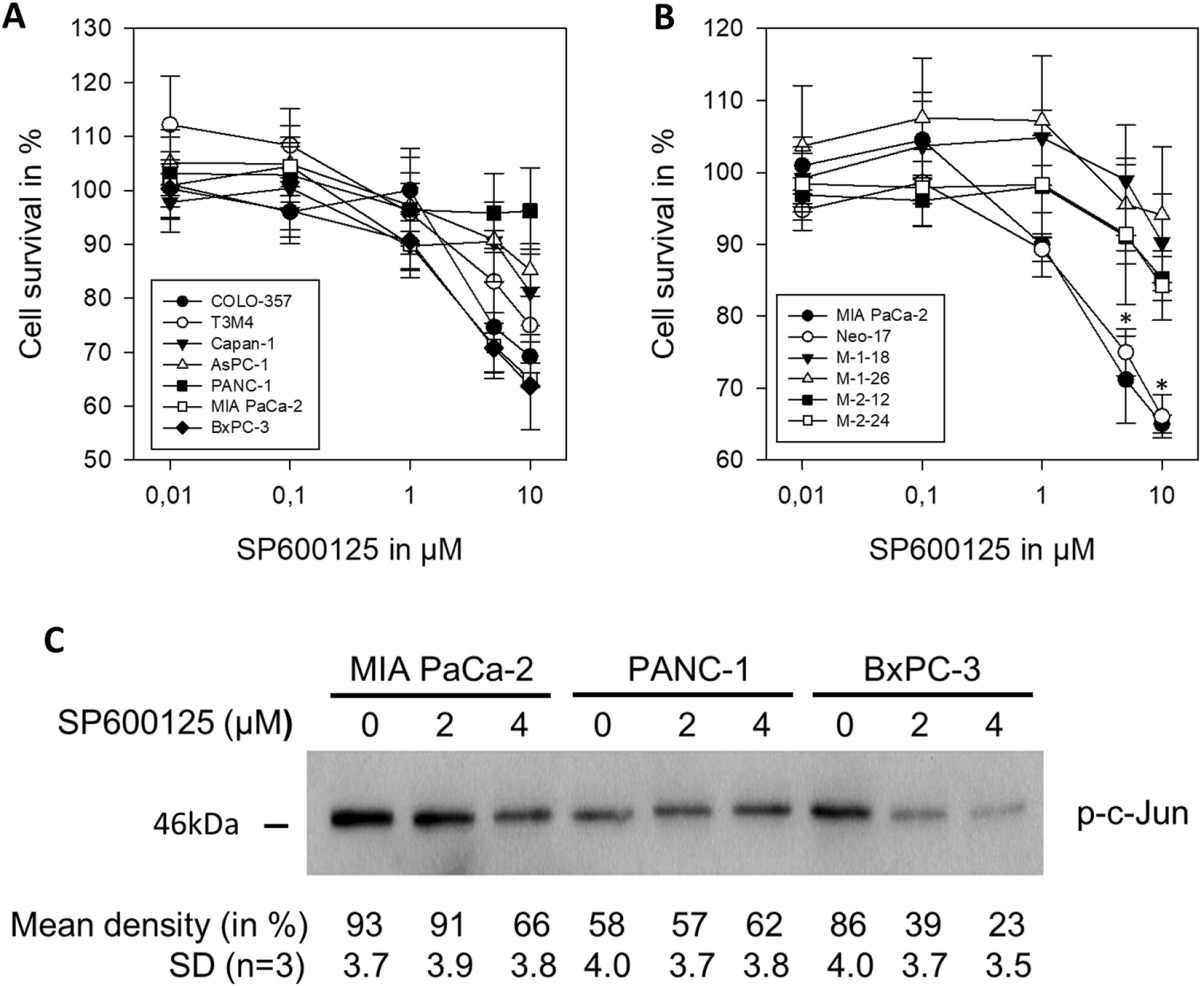
Pharmacological JNK inhibition and the effect on pancreatic cancer cell growth. **A**, **B** Effect of the JNK inhibitor SP600125 on basal cell proliferation in WT cell lines (**A**) and after JNK KD (**B**). Indicated cells (10,000 cells/well) were cultured for 24 h in 96-well plates in complete medium followed by another 48 h in the absence or presence of increasing concentrations of SP600125 followed by the MTT assay. Results are shown as means (±SD) from three separate experiments with quadruplicate determinations compared to untreated control (DMSO only). **B** Cell proliferation was significantly inhibited at 10 µM SP600125 in wild-type (●) and Neo-17 (o). (C) Reduced JNK activity by SP600125 displayed by phosphorylated c-Jun. SP600125 markedly reduced JNK activity in MIA PaCa-2 and BxPC-3 cells while it was without effect in PANC-1. A representative blot is shown of three independent experiments. *p < 0.05 compared to JNK1 and JNK2 KD cells.

**Fig. 3:**
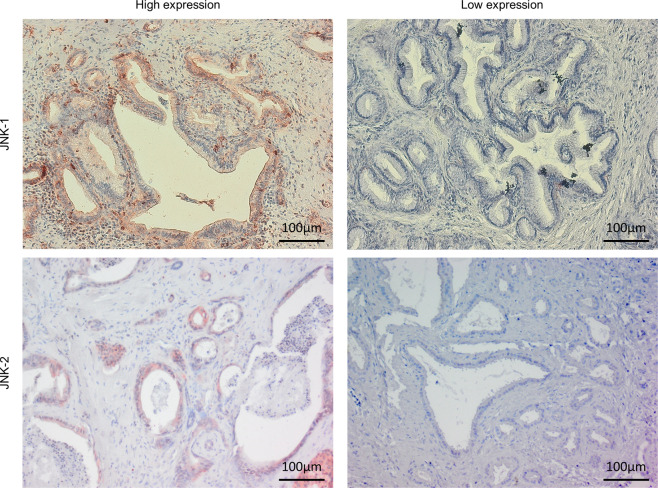
JNK1 and JNK2 expression in human pancreatic cancer tissue. Resected pancreatic cancer specimens were immunohistochemically stained for JNK1 and 2 expressions (JNK1: ab110724, JNK2: ab76125). Examples for high expression are shown on the left, low expression on the right. Scale bar: 100 µm.

All seven tested human pancreatic cancer cell lines expressed higher levels of JNK1 and lower levels of JNK2 protein at various levels (Fig. 1A). JNK activity was present in all cell lines with the highest levels in MIA PaCa-2, PANC-1, and BxPC-3 (Fig. 1A). Incubation of cells in the presence of the specific pan-JNK inhibitor SP600125 resulted in a dose-dependent reduction of cell growth (Fig. 2A). The growth inhibitory effects of SP600125 correlated well with the basal pan-JNK activity with MIA PaCa-2 (7.9 relative JNK activity) and BxPC-3 (7.4) being most sensitive at 10 µM except for PANC-1 (6.4). SP600125 did not exert any inhibitory effect at non-toxic concentrations ≤10 µM in PANC-1 (Fig. 2A, C). Immunohistochemistry analysis of JNK expression in the screened resected specimen showed highly variant expression with expression patterns ranging from nearly absent expression to very high expression in most of the malignant cells (Fig. 3).

### Selective inactivation of endogenous JNK1 and JNK2 expression and its effect on cell proliferation

In order to determine selective functions of JNK1 and JNK2 in pancreatic cancer cells, MIA PaCa-2, with highest JNK activity and high levels of JNK1 and 2 protein, as well as PANC-1, with high JNK expression but lacking response to pharmacological JNK targeting, were used (Fig. 1A). Cell clones were established expressing shRNAs targeting either JNK1 or JNK2. In MIA PaCa-2, a highly specific knockdown (KD) was achieved in M-1–18 and M-1–26 (JNK1 KD) and M-2–12 and M-2–24 (JNK2 KD) (Fig. 1C). In PANC-1, KD of JNK2 was specific without affecting JNK1 expression, while cells expressing shRNA targeting JNK1 showed double-KDs of both isoforms (Fig. 1B). Single KD of JNK1 in MIA PaCa-2 reduced total JNK activity, whereas inhibition of JNK2 protein was without marked effect on total JNK activity, possibly due to the relatively low level of JNK2 and the high level of JNK1 responsible for total JNK protein in MIA PaCa-2 (Fig. 1D).

**Fig. 4:**
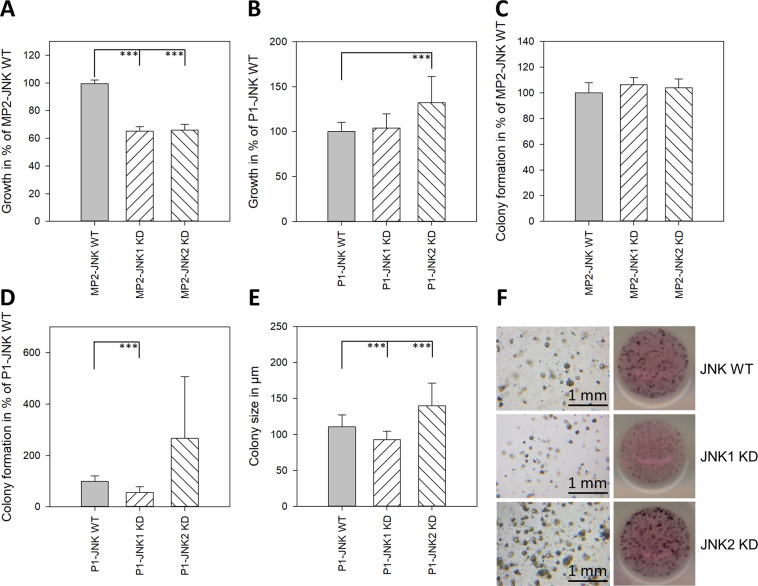
Effect of JNK KD on cell proliferation and colony formation. **A**, **B** Effect of JNK down-regulation on basal cell proliferation. Indicated cells (10,000 cells/well for MIA PaCa-2, 5000 cells/well for PANC-1) were cultured for 48 h in 96-well plates in complete medium and analyzed by the MTT assay. Results are shown as optical density and are means (±SD) from three separate experiments with quadruplicate determinations. **C**–**F** Anchorage-independent growth. MIA PaCa-2 was not influenced by JNK downregulation (C). JNK2 KD in PANC-1 increased colony formation and size while JNK1 KD showed opposing effects (**D**, **E**). Representative images of PANC-1 colony size (left, 2.5× magnification) and colony formation (right, scanned image after MTT staining) are shown in (**F**). Scale bar: 1 mm. Data are shown as means of three independent experiments of triplicate determinations ±SD. JNK WT cells (WT and Neo clones), JNK1 KD clones, and JNK2 KD clones were analyzed together. ***p < 0.001 compared to JNK WT cells.

In MIA PaCA-2, inhibition of JNK1 and JNK2 similarly reduced anchorage-dependent cell growth by 35% (JNK1) and 34% (JNK2) and was without effect on anchorage-independent colony formation (Fig. 4A, C). However, in PANC-1, the results were more differentiated. While the double KD of JNK1 and -2 did not alternate anchorage-dependant cell growth, JNK2 KD significantly increased cell growth by 32% (Fig. 4B). Anchorage-independent colony formation underlined these differences. JNK2 KD impressively increased colony formation by 166% and size by 26%, while JNK1 KD reduced colony formation compared to JNK WT cells by 44% (Fig. 4D, E). The effect did not depend on total JNK activity only (Fig. 1B). MIA PaCa-2 clones were also incubated with the pharmacological JNK inhibitor. SP600125 (10 µM) completely lost its inhibitory effect on cell proliferation in JNK1 shRNA expressing clones M-1–18 and M-1–28 at nontoxic concentrations, while the inhibitory effect was less pronounced in JNK2 shRNA expressing clones M-2–12 and M-2–24 compared to wild-type and sham-transfected cells (M-Neo-17) (Fig. 2B).

### Effects of JNK1 and JNK2 inhibition on cell motility, and invasion

**Fig. 5:**
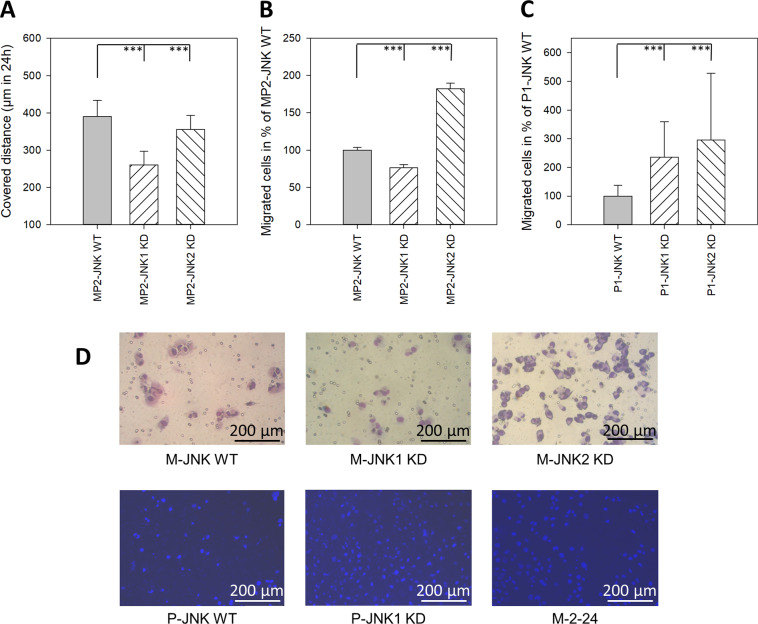
Effect of JNK downregulation on cell movement invasion. **A** Single-cell movement. The total distance (μm in 24 h) of individual cells (*n* = 30) covered within 24 h was evaluated using the ImageJ 1.32 and Simple Track program. Results are shown as the mean distance (±SD) in μm covered within 24 h. **B**, **C** In vitro invasion in the Boyden chamber assay. Indicated cells were seeded into the upper chamber and the number of cells that managed to invade the lower side of the chamber through 8 µm pores was determined after 36 h. **B**, **C** Migrated cells in % of JNK WT cells. Results are shown as mean (±SD) number of migrated cells in % of JNK WT cells within 36 h of three separate experiments. **D** Representative examples of the Boyden chamber assay. JNK WT cells (WT and Neo clones), JNK1 KD clones, and JNK2 KD clones were analyzed together. ***p < 0.001 compared to JNK WT cells.

Nondirected cell movement was measured in MIA PaCa-2 using single-cell movement over 24 h. Here, the movement was reduced in the KD of either isoform from 390 µm ± 43 µm SD in JNK WT cells to 261 µm ± 37 µm SD (JNK1 KD) and 365 µm ± 37 µm SD (JNK2 KD), respectively (Fig. 5A). Alongside that, in the directed invasion assay, the Boyden chamber revealed a marked inhibition of cell migration in JNK1 KD clones of MIA PaCa-2 by 24% (Fig. 5B).

Surprisingly, cell migration was dramatically enhanced in all clones showing reduced JNK2 expression. This applies to MP2-JNK2 KD clones (+92%) as well as all 4 clones in PANC-1 (single JNK2 KD (+195%), as well as double KD (+135%)) (Fig. 5B, C).

### JNK1 and -2 and markers of epithelial-to-mesenchymal transition (EMT)

**Fig. 6:**
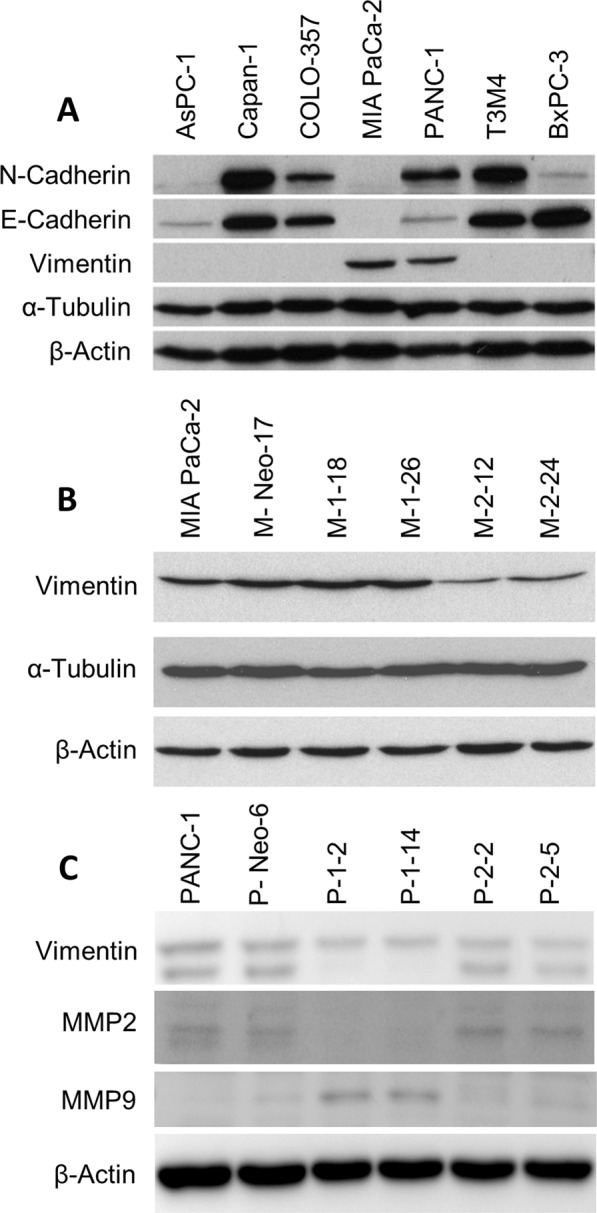
EMT markers in dependency of JNK expression. **A** Immunoblot analysis of N-Cadherin, E-Cadherin, Vimentin, and α-Tubulin in pancreatic cancer cell lines. β-Actin is used to confirm equal loading. **B**, **C** JNK2 downregulation leads to reduced Vimentin expression. MMP9 is upregulated in the KD of both isoforms, but predominantly in JNK1 KD while MMP2 expression is lost under JNK1 KD.

Due to the marked effects on cell migration, the expression levels of EMT-markers were determined. These included the main cytoskeleton and cell adhesion proteins N-cadherin, E-cadherin, Vimentin, and α-Tubulin (Fig. 6A). Only our target cell line MIA PaCa-2 and PANC-1 showed detectable levels of Vimentin. Furthermore, MIA PaCa-2 showed no E-cadherin expression and minimal N-Cadherin expression.

While α-tubulin expression in MIA PaCa-2 was not altered, Vimentin protein expression was downregulated in clones with reduced JNK2 expression in both cell lines (Fig. 6B, C). Overall matrix-metalloproteinase (MMP) expression was low in PANC-1. JNK1 KD cells lost expression of MMP2, but at the same time showed upregulation of MMP9 expression. MMP9 expression was hardly detectable in JNK WT cells and showed faint upregulation in JNK2 KD cells in vitro as well (Fig. 6C).

### Effects of JNK1 and JNK2 inhibition on orthotopic xenograft formation

**Fig. 7:**
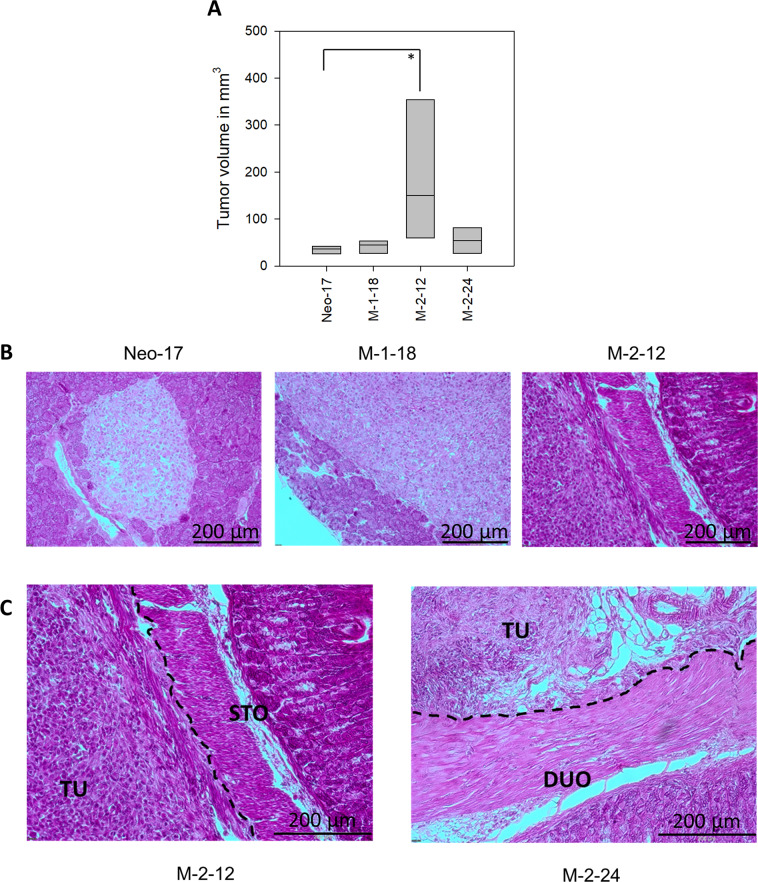
Orthotopic in vivo xenograft. **A** Tumor volume. JNK2 KD cells showed increased tumor volume, especially for M-2–12. **B**, **C** H.E. staining revealed solid pancreatic tumors in all xenografts. In control cells and JNK1 KD cells (M-1–18), the tumor was restricted to intrapancreatic growth while JNK2 KD clones regularly infiltrated surrounding organs. Tumors (TU) of M-2–12 (left) and M-2–24 (right) showed infiltration of the stomach (STO) and duodenum (DUO). The dashed line represents the tumor infiltrative line. This infiltrative pattern was not seen in control cells and JNK1 downregulated cells. Scale bar: 200 µm. *p < 0.05.

**Fig. 8:**
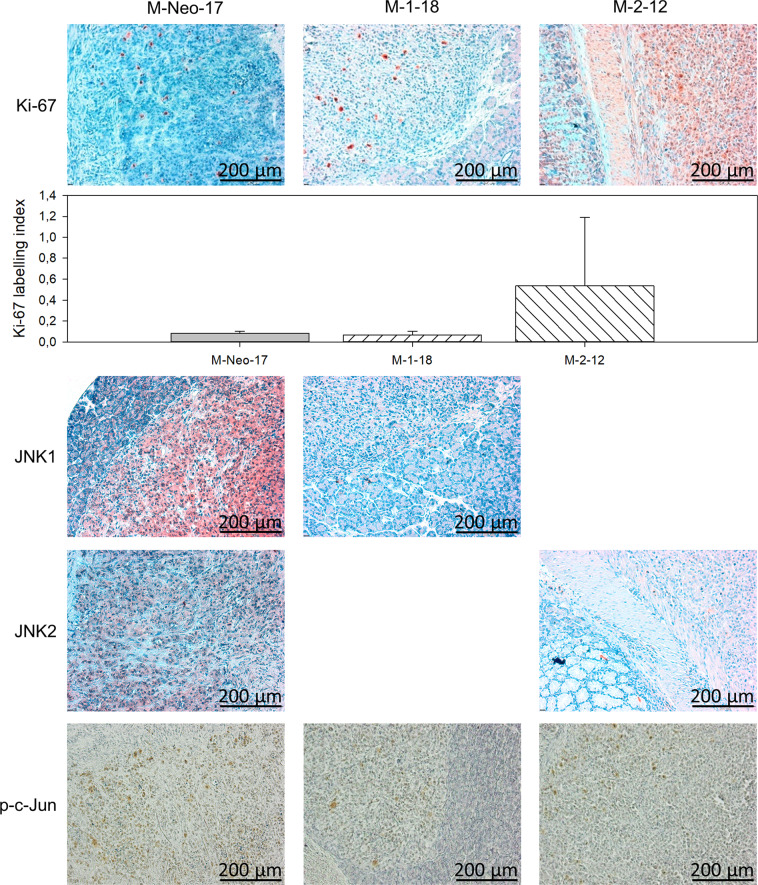
Immunohistochemical analysis of xenograft tumors. Ki-67 staining corresponds to tumor volume with high staining in JNK2 KD clones. Ki-67 positivity score was counted in three sections per tumor and was displayed as means (±SD) of three sections: JNK WT: 8.2% pos., JNK1 KD: 6.6% pos., JKN2 KD: 53.5% pos. (first two lanes). Isoform KD was retained in vivo with reduced expression of either protein in the target clones compared to M-Neo-17. KD of either isoform reduced p–c-Jun levels in vivo (lanes 3–5).

In order to investigate the possible effects of JNK1 and JNK2 on xenograft formation, an orthotopic mouse model was used [19]. The focus of the xenograft model was to investigate the infiltrating potential of JNK2-shRNA expressing clones in vivo. To minimize the number of animals, only Neo-17 (shRNA expressing control) as the control group (*n* = 10) and M-1-18 (JNK1-shRNA expressing clone, *n* = 7) were used and compared with two JNK2-shRNA expressing clones (M-2–12, M-2–24, each *n* = 7). Two animals (2/31, 6.5%) did not survive the surgical procedure and one animal died 1 day after surgery. The remaining 28 animals were followed up and evaluated for tumor formation and growth. Weight evaluation displayed no difference among the groups. Twenty mice had readily visible pancreatic tumors at the postmortal section. One small tumor was only found microscopically (Neo-17). JNK2 shRNA expressing clones (M-2–12 and M-2–24; 11 (11 out of 13) tended to establish orthotopic pancreatic tumors more often than Neo-17 (4 out of 10; Fisher Exact test: *p* = 0.039). In addition, tumors of JNK2 shRNA expressing clones, especially M-2–12 with 186 ± 58 mm^3^ SE, were larger in volume than Neo-17 tumors 35 ± 3 mm^3^ SE (*p* < 0.05) (Fig. 7A). The enhanced in vivo growth of JNK2-KD clones was associated with increased proliferation as seen in Ki-67 staining and Ki-67 labeling index (Fig. 8).

The results of the H.E. staining revealed solid intra-pancreatic tumors for all cell clones (Fig. 7B). All tumors formed by Neo-17 and also M-1-18 were well restricted to the pancreas and did not show any signs of infiltration. In contrast, tumors of M-2-12 and M-2-24 invaded adjacent organs and infiltrated the duodenum and the stomach (Fig. 7C). The invasion was detected in 8 out of 11 JNK2 shRNA expressing tumors but in none of the Neo-17 control (0/4) and JNK2 shRNA expressing M-1–18 (0/4) tumors. Compared to control, the in vivo infiltrating potential was increased after JNK2 inhibition (*p* = 0.026, Fisher’s exact test).

**Fig. 9:**
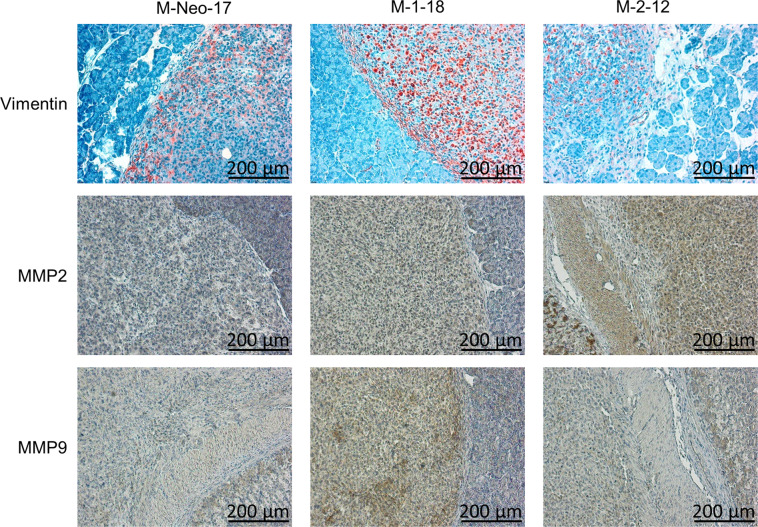
EMT markers in vivo. Vimentin expression is strongly reduced after JNK2 KD but retained after JNK1 KD in vivo (first lane). MMP expression in JNK WT cells is generally low. MMP2 expression is upregulated after KD of both isoforms, but predominantly after JNK2 KD, while MMP9 expression is increased after JNK1 KD (lanes 2 and 3). Scale bar: 200 µm).

We confirmed the stable reduction of isoform expression in vivo. This was associated with reduced phosphorylation of JNK target gene c-Jun in both isoforms (Fig. 9).

Interestingly, the previously described reduced expression of Vimentin was also confirmed in vivo. The highly invasive tumors of M-2–12 and M-2–24 showed drastically reduced Vimentin expression as shown in the immunohistochemistry analysis (Fig. 9). JNK WT cells also showed little MMP staining in vivo, matching the in vitro results. On the other hand, MMP upregulation was more prominent in vivo, especially with strong staining of MMP2 in highly invasive JNK2 KD clones. Furthermore, the upregulation of MMP9 expression was confirmed in JNK1 KD cells in vivo (Fig. 9).

## Discussion

JNKs play important roles in mediating several biological functions involved in altering the malignant phenotype of cancer cells via modulation of the Ras-pathway and p53 functions [5, 8]. Human pancreatic cancer is a devastating disease characterized by strong local invasion, stromal fibrosis, local recurrence, and metastases and frequently harboring Ras- and p53-mutations [4]. Recently, pancreatic cancer progression on various levels was associated with JNK activation [5, 8, 14, 20–22]. However, none of these studies selectively examined the roles of the different JNK-isoforms.

In the present study, this issue was addressed by KD of either JNK-1 or -2. Thereby, we were able to show that cultured human pancreatic cancer cells express both JNK1 and JNK2 and display various levels of JNK activity. In all cell lines, JNK1 was the predominant isoform, both regarding protein expression as well as overall kinase activity. Cell proliferation was inhibited in the presence of SP600125, a selective JNK inhibitor, at nontoxic concentrations (≤10 µm), and the effect correlated well with the level of total JNK activity in each cell line. Interestingly, although PANC-1 showed a relatively high overall JNK-activity, SP600125 did not exert growth reducing effects. In this context, we were able to show that SP600125 did not influence the overall JNK activity in PANC-1. Here, Recio-Boiles et al. [23] also confirmed little response of PANC-1 to SP600125 while the alternative JNK inhibitor JNK-INH 8 lead to reduced anchorage-independent growth of PANC-1, and by that suggesting intrinsic resistance toward SP600125, but supporting our findings. Immunohistochemistry also demonstrated JNK activity in human pancreatic cancer tissues, confirming the clinical significance. A broad range immunohistochemical study, separately analyzing JNK1 and 2 expressions in the resected specimen is currently running and are going to be evaluated separately.

In order to investigate possible specific roles of JNK1 and -2, cell clones were generated using shRNA transfection targeting JNK1 and JNK2. MIA PaCa-2 cells were chosen due to their high basal JNK activity and relatively high JNK1 and -2 protein levels in comparison to the other cell lines, while PANC-1 was chosen due to its high JNK activity but lacking pharmacological response. Specific knockdown experiments in human fibroblasts revealed that inhibition of JNK1 inhibited proliferation, while inhibition of JNK2 increased proliferation by augmenting c-Jun levels [24]. JNK KD in MIA PaCA-2 consistently recapitulated the response to SP60125 with reduced cell anchorage-dependent cell growth. Interestingly, although showing resistance towards SP600125, PANC-1 response to JNK KD was more prominent and predominantly seemed to be influenced by JNK2. Specific JNK2 KD not only significantly increased anchorage-dependent cell growth, but also increased colony formation and colony size. PANC-1 clones with double KD of JNK1 and -2 did not differ in the MTT assay, and not only lost the higher colony formation ability of single JNK2 KD but even showed fewer colonies than JNK WT cells. Our observations align with previous findings in human fibroblasts, suggesting opposing functions of both JNK isoforms on cell growth, however, cell line-specific characteristics still influence the final phenotype.

Furthermore, we were now able to study the distinct role of JNK1 and JNK2 in the migration and invasion of pancreatic cancer cells for the first time. Screening of non-directed single-cell movement revealed strong reduction by JNK1 KD as well as reduced movement in JNK2 KD cells. Invasion in the Boyden chamber assay seems to be predominantly influenced by JNK2 KD, similar to the colony formation assay. Single JNK1 KD in MIA PaCa-2 reduced invasion similarly to the single-cell movement data. A comparable role for JNK1 was described in fish keratinocytes and rat bladder tumor epithelial cells [25]. The pro-migratory role of JNK1 was also demonstrated after KD in multiple carcinoma cell lines indicating a major role in cell mobility [26].

In contrast to the pro-migratory role of JNK1, our results suggest an inhibiting function for JNK2 on cell invasion. Downregulation of JNK2 protein was associated with a dramatic enhancement of cell migration in the Boyden-chamber assay. We argue, that the increased invasion of PANC-1 clones expressing shRNA targeting primarily JNK1 results in the reduced expression of JNK2. The difference to single JNK2 KD cells might be caused by the lack of pro-migratory signals from JNK1 in these double KD clones.

To investigate possible effects on in vivo tumor growth an orthotopic xenograft model was chosen [19]. The orthotopic model as cell line-derived xenograft allows for better investigation infiltration and migration compared to the subcutaneous model [27, 28]. Macroscopically, no distant metastases were observed. No peritoneal spread was seen demonstrating that the injection of tumor cells into the pancreas was of high quality without leakage [28]. No marked effects were noticed for JNK1 downregulated clones compared to controls. The results of the Boyden chamber assay for JNK2 downregulation were confirmed by the in vivo model. Invasion of neighboring organs like duodenum and stomach was only but regularly observed in tumors of JNK2 shRNA expressing cells. Interestingly, while M-2–24 still showed slight JNK2 expression, in M-2–12 a nearly complete knockout of JNK2 was achieved. In vivo, M-2–12 showed significantly larger tumors compared to control transfected clones without higher invasiveness. We speculate that with a tumor-suppressing role of JNK2 in vivo, a nearly complete knockout may potentiate the seen effects on in vivo growth compared to the KD observed in M-2–24.

The reasons for the increased migratory potential after JNK2 inhibition were so far unknown. In order to study these differences, we focused our studies on markers of EMT. As demonstrated in Fig. 6, some pancreatic cancer cell lines endogenously express Vimentin, especially our target cell lines MIA PaCa-2 and PANC-1, cell lines with an epithelial-mesenchymal phenotype. Vimentin expression in epithelial cells is commonly gained during EMT, which is a hallmark of malignant transformation [29]. Furthermore, EMT is essential for tumor progression toward a metastatic state and Vimentin expression was associated with tumor progression and invasion in breast and lung cancer [30–32]. Interestingly, Vimentin KD also reduced the expression of proteins involved in focal adhesion complexes [31].

In our study, analysis of JNK1 and JNK2 downregulated clones revealed no effect on Actin or Tubulin expression. However, inhibition of JNK2 resulted in a downregulation of Vimentin protein in vitro and in vivo. To our knowledge, this is the first description of reduced Vimentin expression paralleled by phenotype with enhanced invasion and migration. The reasons for this are still unclear. We propose the cells to be more flexible and by that increase their migratory potential. Furthermore, the loss of adhesion molecules may participate in facilitated invasion. In addition, alterations in MMP expression plays an important role in invasion. Especially the gelatinases MMP2 and MMP9 are able to digest Collagen IV, a major component of the basement membrane [33]. MMP2 and −9 deficient mice showed slowed tumor progression and metastases [34]. While MMP expression in vitro is low, especially MMP2 expression in highly invasive JNK2 KD cells is increased in vivo, offering a possible explanation for increased invasiveness. However, MMP9 upregulation was consistently observed in vitro and in vivo in JNK1 KD cells without an obvious phenotype.

Previously, many reports have suggested that JNK1 and JNK2 fulfill many overlapping and redundant functions [11]. However, our results suggest that especially JNK2 exerts tumor-suppressing functions in human pancreatic cancer and is opposed by JNK1. To our knowledge, this is the first report demonstrating opposing functions for JNK1 and JNK2, especially for migratory functions.

Therefore, JNKs may be an attractive target for cancer treatment due to their effects on proliferation, movement, and migration. However, further studies are necessary to unravel the specific functions of JNK1 and JNK2. In the future, specific targeting of the c-Jun N-terminal kinase pathway may be of potential therapeutic interest, especially in patients with high c-Jun activity but lacking a strong JNK2 activation.
